# Control of tuberculosis in large cities in developed countries: an organizational problem

**DOI:** 10.1186/1741-7015-9-127

**Published:** 2011-11-28

**Authors:** Joan A Caylà, Angels Orcau

**Affiliations:** 1 Epidemiology Service, Public Health Agency of Barcelona, Pl. Lesseps, 1, 08023, Barcelona, Spain

## Abstract

Tuberculosis (TB) is still a serious public health issue, even in large cities in developed countries. Control of this old disease is based on complicated programs that require completion of long treatments and contact tracing. In an accompanying research article published in *BMC Public Health*, Bothamley and colleagues found that areas with a ratio lower than one nurse per forty notifications had increased rates with respect to TB notifications, smear-positive cases, loss to follow-up and treatment abandonment across the UK. Furthermore, in these areas there was less opportunity for directly observed therapy, assistance with complex needs, educational outreach and new-entrant screening. In this commentary, we discuss the importance of improving organizational aspects and evaluating TB control programs. According to Bothamley and colleagues, a ratio of one nurse per forty notifications is an effective method of reducing the high TB incidences observed in London and in other cities in developed countries, or to maintain the decline in incidence in cities with lower incidences. It is crucial to evaluate TB programs every year to detect gaps early.

See related article: http://www.biomedcentral.com/1471-2458/11/896

## Introduction

**Figure 1:**
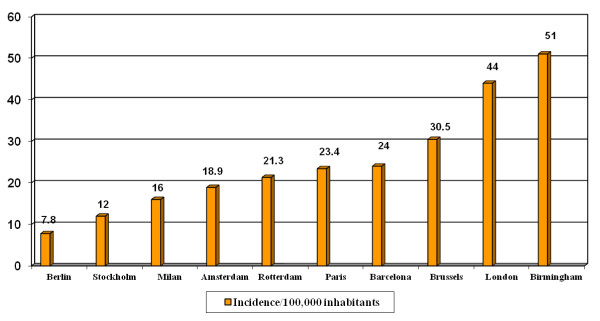
**Incidence of tuberculosis in selected European Union cities, 2009**. Incidence of this disease in cities of low-incidence European Union countries showing high level of incidence in several urban areas. Source: references [1-3].

Tuberculosis (TB) is a contagious bacterial disease that can be fatal without treatment, and any delay in diagnosis of pulmonary and laryngeal cases increases the chances of transmission. The incidence of TB is greater in large cities than in non-urban areas or in the country as a whole, as Bothamley and colleagues describe in their article published this month in *BMC Public Health *[1]. The authors also found that cities that did not achieve the target of one nurse per forty TB notifications had worse TB control indicators than cities reaching this target. The authors concluded that control depends on adequate numbers of specialist TB nurses for early detection and case holding. They also observed strikingly high incidences in 2009 in cities such as Manchester (59.1/100, 000) and London (44.4/100, 000) in relation to other English cities. In other western European cities, in general, the incidences were also lower [2,3] (Figure 1). These data led to London appearing widely in the media as 'the European capital of TB' [4] following publication of a paper in *The Lancet *with the subtitle 'London has one of the highest rates of TB in Western Europe, and the city homeless population are most at risk and the hardest to treat' [5].

## Discussion

TB affects the most vulnerable populations, including HIV-infected people, drug abusers, the homeless and immigrants, in a disproportionate way. These populations mostly live in urban settings and, as such, influence TB epidemiology in large developed cities [6,7]. TB control programs have to adapt to any new challenge, and new control strategies should be implemented when a new problem arises [8]. New York City, for instance, had to deal with a serious epidemiological situation when the AIDS epidemic broke out. From 1978 to 1992, the number of patients with TB nearly tripled due to HIV infection, drug resistances and the abandonment of TB programs, but fortunately they were able to apply comprehensive control measures (including directly observed therapy (DOT) and control of nosocomial transmission) and the situation reversed [9]. The peak of incidence was observed in 1992 (3, 811 cases, incidence of 52.0/100, 000), declining to 10.8/100, 000 (895 cases) in 2008 [10]. TB was considered a political priority and the New York TB program received substantial funds; DOT was the rule with a high number of DOT workers. The use of incentives for patients dually infected with TB and HIV (the equivalent of $100/month for adherent patients) or a $25 'show-up voucher' to inmates to encourage them to continue their TB treatment after release from prison, along with other measures to elevate the status of DOT workers and recognize them as the true heroes of modern public health, contributed to the effectiveness of the program [11].

With the financial crisis, the New York model is difficult to replicate in most cities due to its high cost and TB programs should include new control strategies based on improvements in health organization. Solid public health organization, combined with precise knowledge of local healthcare and the social system, along with the co-operation of hospitals and specialized clinics, could help to better implement specific control strategies.

**Figure 2:**
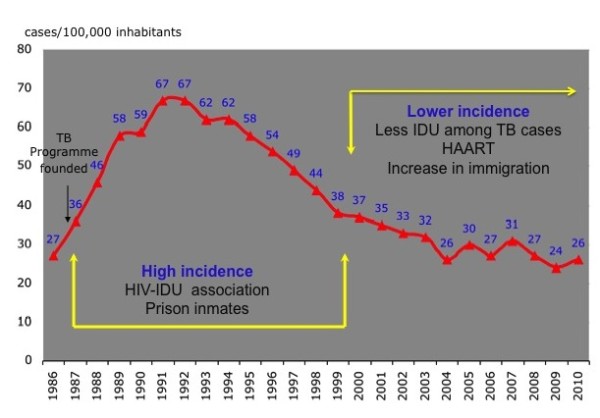
**The evolution of tuberculosis in Barcelona, 1986-2010**. The graph shows tuberculosis cases per 100, 000 inhabitants over time. During the 1990s a high incidence period was observed, mostly due to HIV-infected injecting drug users. With the implementation of control measures and the generalization of antiretroviral treatments, the annual decline was about 10%. This decline was attenuated during the low incidence period due to massive immigration from high burden countries. TB, tuberculosis; IDU, injecting drug users; HAART, highly active antiretroviral therapy.

Unfortunately, in many cities it is very difficult to create or maintain a team of nurses dedicated solely to TB due to relatively low TB incidence and also because of limited resources dedicated to this disease. For example in Barcelona, the TB Program has had, from its inception in 1987, public health nurses (PHN) who carry out follow-up with patients and perform contact tracing but are also in charge of other communicable diseases. The program also involves active surveillance of cases (reporting of cases is promoted, microbiological results and hospital discharges are monitored, and the link between AIDS and TB registers is also monitored). In the first few years, we observed the influence on TB epidemiology of injecting drug users (IDU) and persons with HIV infection, and incidence increased until it peaked in 1992 (1, 096 cases, 67/100, 000) [12]. DOT was added early for those patients with predictors of poor adherence (homeless, IDU, prisoners). The inclusion of DOT in methadone programs and tight coordination between the TB programs in the prisons and the city was very useful in achieving a high level of treatment completion among IDU [13]. Following the increase use of DOT and the use of antiretroviral treatments, TB incidence decreased, but with the massive immigration observed from 2000 onwards, this decline has been attenuated (Figure 2). Consequently, since 2003 we have incorporated community health workers (CHW) to help PHN in follow-up and contact tracing. These CHW also visit the patient at home, at the hospital, at the DOT facility and act as translators and cultural mediators [14].

**Figure 3:**
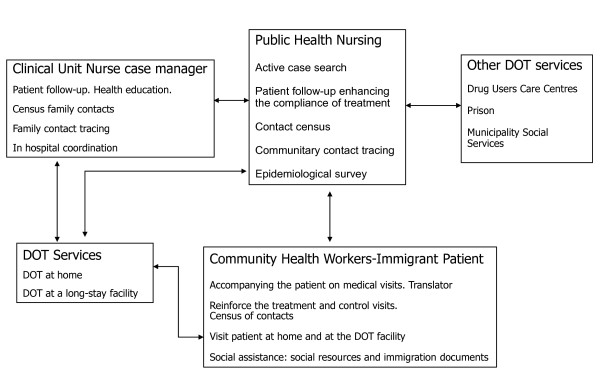
**Barcelona Tuberculosis Program: organizational aspects**. Network developed and relationships among different providers for tuberculosis control in Barcelona. DOT, directly observed treatment.

The link between surveillance, control and operational research has always been a priority, and has facilitated coordination among all health workers involved in TB control (clinicians, microbiologists, epidemiologists, social services managers). In the last few years, TB care services have been reorganized due to massive immigration and to concentrate the contact tracing in five TB Units. All large hospitals in the city have TB clinical units that carry out diagnosis, treatment, monitoring of the patient and contact tracing in the household contacts. The TB case manager nurse in these clinical units is the key professional in this reorganization. Figure 3 outlines the organization of TB care in Barcelona. The city is divided into four health areas and in each area there is an integrated TB working committee which is representative of all health workers involved in TB control. The TB Program team includes a physician, 12 PHN for about 500 cases a year (ratio 1/35-45) and 6 CHW. Both PHN and CHW are also in charge of other notifiable diseases (TB represents about 40% of the total workload). Close coordination with the Unit Clinic case manager nurse allows the management of cases. The CHW time contribution is presently based on immigrant TB patient characteristics (number of cases and country of origin).

It is important to evaluate TB programs every year in order to identify gaps in services, data monitoring, and so on. The audit of TB services against elements contained in the national TB Action Plan (Table 5 of the accompanying paper) is a very good example as it can detect which criterion needs to be improved in each city. The epidemiological evaluation of a TB program should be based on few indicators in order to facilitate annual evaluations (decline in TB incidence, diagnostic delay, completion of TB treatment, coverage of contact tracing, TB meningitis in children under 4 years) [12].

Over the next few years, with a probable decline in TB incidence, it may be difficult to maintain big teams of specialized TB nurses within the public health structures. Should this prove to be the case, the challenge of containing TB in hard-to-reach groups would be daunting and diagnostic delay would be likely to increase in a context of low-incidence and low-resource settings with the possibility of epidemic outbreaks among affected children [15]. The recommendations of Bothamley and colleagues in relation to TB nurses, along with those of scientific societies published since 1988 [16], could be helpful in maintaining these resources because a ratio of specialized TB nurses to patients is established. In large cities, maintaining the TB program in a Public Health structure that also performs surveillance and control of other notifiable diseases and epidemic outbreaks could be crucial in achieving long-term resource and management expertise.

## Conclusions

The current economic crisis will have long-term impacts on communicable disease control and it is critical to keep the public health budget [17] at an adequate level, although TB programs can increase their efficiency by improving their organizational structures. To take advantage of other resources for transmissible diseases, such as the resources for HIV prevention and for control of epidemic outbreaks, co-operation with other programs (including Immigration and IDU services) will be essential. Brigs stated in 1914 that 'Public health is purchasable; a community can determine its own death rate' [18]. Following these ideas, Reichman strongly recommends fostering and maintaining the political will that allows enhancement of public health programs that can move towards the elimination of TB [19]. Each city needs to define its needs according to its particular epidemiological situation, but the ratio of one nurse per forty TB cases, as suggested by Bothamley and colleagues, is a very good starting point.

## List of abbreviations

TB: tuberculosis; DOT: directly observed therapy; PHN: public health nurses; CHW: community health workers; IDU: injecting drug users.

## Competing interests

The authors declare that they have no competing interests.

## Authors' contributions

Both authors contributed equally to the production of the manuscript and also read and approved the final manuscript.

## Authors' information

AO and JAC are working in the Barcelona TB programs and each year organize an International TB Workshop. AO coordinates the PHN and CHW programe, analyzes the TB data base and writes an annual report.

JAC coordinates the Barcelona TB Investigation Unit and is the principal investigator at site 31 of the CDC TB Trials Consortium and of TB Research at the Spanish Society of Pneumology (SEPAR).

## Pre-publication history

The pre-publication history for this paper can be accessed here:

http://www.biomedcentral.com/1741-7015/9/127/prepub
